# Synthesis of Novel Tamarind Gum-*co*-poly(acrylamidoglycolic acid)-Based pH Responsive Semi-IPN Hydrogels and Their Ag Nanocomposites for Controlled Release of Chemotherapeutics and Inactivation of Multi-Drug-Resistant Bacteria

**DOI:** 10.3390/gels7040237

**Published:** 2021-11-27

**Authors:** Kasula Nagaraja, Kummari S. V. Krishna Rao, Sunmi Zo, Sung Soo Han, Kummara Madhususdana Rao

**Affiliations:** 1 Polymer Biomaterial Design and Synthesis Laboratory, Department of Chemistry, Yogi Vemana University, Kadapa 516005, Andhra Pradesh, India; nagarajakasula33@gmail.com; 2 School of Chemical Engineering, Yeungnam University, 280 Daehak-ro, Gyeongsan 38541, Gyeongbuk, Korea; sunmizo@ynu.ac.kr (S.Z.); sshan@yu.ac.kr (S.S.H.); 3 Research Institute of Cell Culture, Yeungnam University, 280 Daehak-ro, Gyeongsan 38541, Gyeongbuk, Korea

**Keywords:** tamarind gum, hydrogels, semi-IPNs, green synthesis, silver nanoparticles, drug delivery, chemotherapeutics, HCT116 cell, anti-microbial

## Abstract

In this paper, novel pH-responsive, semi-interpenetrating polymer hydrogels based on tamarind gum-*co*-poly(acrylamidoglycolic acid) (TMGA) polymers were synthesized using simple free radical polymerization in the presence of bis[2-(methacryloyloxy)ethyl] phosphate as a crosslinker and potassium persulfate as a initiator. In addition, these hydrogels were used as templates for the green synthesis of silver nanoparticles (13.4 ± 3.6 nm in diameter, TMGA-Ag) by using leaf extract of *Teminalia bellirica* as a reducing agent. Swelling kinetics and the equilibrium swelling behavior of the TMGA hydrogels were investigated in various pH environments, and the maximum % of equilibrium swelling behavior observed was 2882 ± 1.2. The synthesized hydrogels and silver nanocomposites were characterized via UV, FTIR, XRD, SEM and TEM. TMGA and TMGA-Ag hydrogels were investigated to study the characteristics of drug delivery and antimicrobial study. Doxorubicin hydrochloride, a chemotherapeutic agent successfully encapsulated with maximum encapsulation efficiency, i.e., 69.20 ± 1.2, was used in in vitro release studies in pH physiological and gastric environments at 37 °C. The drug release behavior was examined with kinetic models such as zero-order, first-order, Higuchi, Hixson Crowell and Korsmeyer–Peppas. These release data were best fitted with the Korsemeyer–Peppas transport mechanism, with *n* = 0.91. The effects of treatment on HCT116 human colon cancer cells were assessed via cell viability and cell cycle analysis. The antimicrobial activity of TMGA-Ag hydrogels was studied against *Staphylococcus aureus* and *Klebsiella pneumonia*. Finally, the results demonstrate that TMGA and TMGA-Ag are promising candidates for anti-cancer drug delivery and the inactivation of pathogenic bacteria, respectively.

## 1. Introduction

Functionalized interpenetrating polymer network (IPN) hydrogels have piqued the interest of researchers as potential materials in a wide range of medical and environmental applications, including affinity chromatography, immobilization technologies, drug-delivery systems and organic dyes and heavy metal ion removal applications, such as ion exchangers, adsorbents, and so on [1,2,3,4,5]. The importance of IPNs is growing due to the numerous opportunities to modify their physico-chemical properties [6]. Natural polymers have successfully replaced a number of synthetic materials, either because the latter were high in cost or because the biodegradable and biocompatible properties of natural polymers outperformed those of synthetic materials [7]. The success of natural polymer hydrogels as biomaterials is mostly due to their resemblance to biological tissues and transformation processes, which allow them to be easily manufactured in a variety of forms and sizes at low manufacturing costs [8]. Natural polymers, on the other hand, have high biocompatibility but poor mechanical qualities; the necessity to preserve biological properties hampers their processing. Hence, to improve the physico-mechanical properties of natural polymer-based hydrogels, they are blended, grafted and embedded with compatible additives [8].

Recently, natural-polymer-based IPN-type hydrogels have been successfully developed for drug delivery, anti-microbial, wound healing, tissue engineering applications [6,7,8]. Banthia et al. developed pH-sensitive polyacrylamide-grafted pectin-based hydrogels for use in the controlled release of salicylic acid. Salicylic drug release was observed to be 86% within 7 h. [9]. Krishna Rao et al. developed pH- and temperature-responsive semi-IPN hydrogel based on pectin and poly(2-dimethylamin)ethyl methacrylate for the controlled drug delivery of 5-fluorouracil. These hydrogels enhanced drug release in various stimuli-responsive environments; finally, 100% drug release was observed within 80 h. These hydrogels could be used for colon-specific drug delivery applications [10]. Gautam Sen et al. developed sesbania gum based on a hydrogel prepared by a microwave-assisted method using acrylamide as a monomer for sustained release applications of 5-flurouracil. These hydrogels enhanced the drug release in alkaline medium [11]. Baljit sing et al. developed poly(vinyl pyrrolidone) and *Azadirachta indica* gum-based hydrogels for the drug delivery of methyl prednisolone. These hydrogels enhanced drug release in phosphate buffer, i.e., 45% prednisolone released in 24 h [12].

Tamarind gum (TMG) is a natural polysaccharide; it is a derivative of seeds of *Tamarindus indica linn*, which is a widely available plant in India and Southeast Asia. TG consists of ‘(1-4)-β-D-glucan residues substituted with α-D-xylopyranose and β-D-galactopyranosyl (1-2)-α-D-xylopyranose linked (1-6) to glucose moieties [13]. TMG is more pH tolerant as well, as it shows significant bioadhesive properties; due to this promising feature, TMG-based materials have been used for drug delivery applications. pH-responsive TMG polysaccharide and sodium alginate blend beads were developed for biotechnology applications [14]. Functionalized carbon-nanotube-incorporated TMG hydrogels were developed for the controlled release of tigecycline [15]. Graphene-oxide-based viscoelastic composite films were developed from blends of poly(vinyl alcohol) and carboxy methyl TMG for the controlled release of ciprofloxacin hydrochloride. The results of these studies indicate that they have anti-microbial characteristics, which helps in human skin keratinocytes [16]. Floating beads of sodium alginate blended with TMG and magnesium stearate were fabricated by the ionotropic gelation technique for the controlled release of risperidone [17]. Simvastatin-based nanoparticles were formulated from polyelectrolyte complexes of chitosan and TMG for anti-cancer applications [18].

Doxorubicin (DOX) is a commonly used chemotherapeutic drug used to treat a variety of cancers, including hematological malignancies and soft tissue sarcomas [19,20,21,22]. It is obtained from a *streptomyces species* and belongs to the anthracycline family, which has potential antibacterial and anticancer action [23,24]. It is an amphiphilic compound comprising a water insoluble aglycone and water-soluble amino sugar functional moiety. DOX’s specific method of action is unknown, although it is thought to bind with DNA via intercalation and block topoisomerase. DOX’s therapeutic applicability is limited due to numerous drug resistance and server-side effects. Furthermore, high dosages of DOX recommended to improve efficiency may have negative side effects on normal tissue cells, particularly those in the kidney and heart. Hence, to overcome the shortcomings of DOX, it was encapsulated or conjugated with polymeric systems [25,26,27].

In the continuation of research on natural-polymer-based functional materials for biomedical applications [28] and based on the above facts, in this study, tamarind gum-*co*-poly(acrylamidoglycolic acid)-based copolymeric semi-IPN hydrogels were composed using bis[2-(methacryloyloxy)ethyl] phosphate as a potential crosslinker by simple free radical polymerization. These hydrogels were studied via FTIR, XRD, SEM and TEM and were employed for the in vitro controlled release of DOX, a chemotherapeutic drug. These hydrogels were employed to study the in vitro cell viability and cell cycle analysis of HCT116 cells. Furthermore, these hydrogels were utilized in the green manufacture of silver nanocomposites, employing aqueous leaf extract of *Teminalia bellirica*.

## 2. Results and Discussion

### 2.1. FTIR Spectroscopy Studies

**Figure 1:**
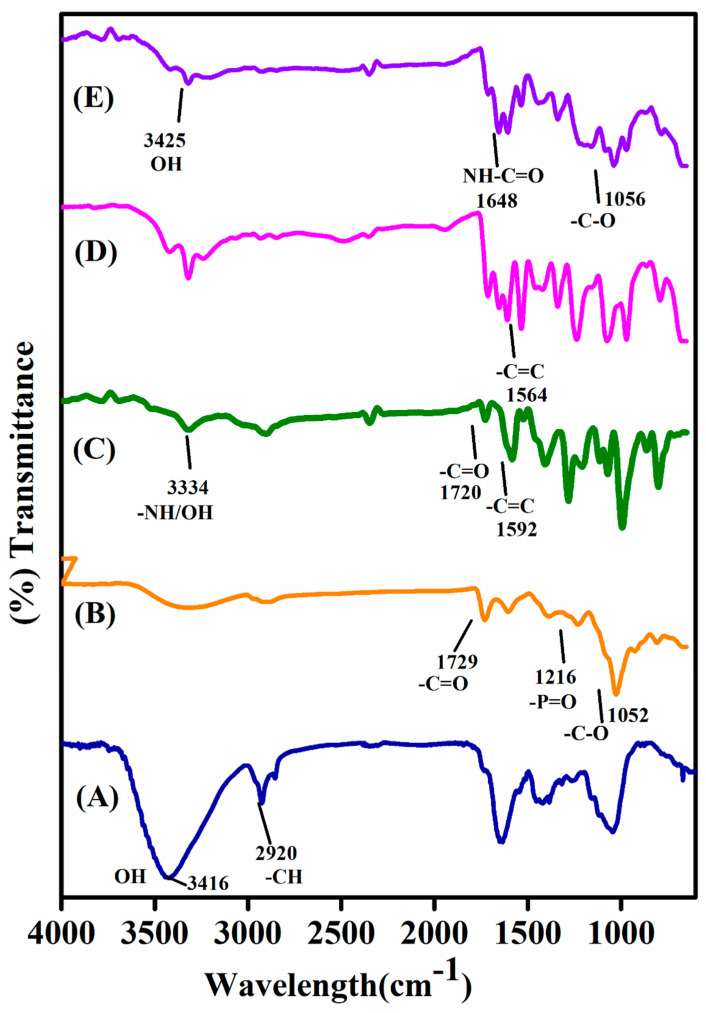
FTIR spectra of TMG polysaccharide (**A**), pristine TMGA hydrogel (**B**), DOX (**C**), DOX loaded TMGA hydrogel (**D**), TMGA-Ag (**E**).

**Figure 2:**
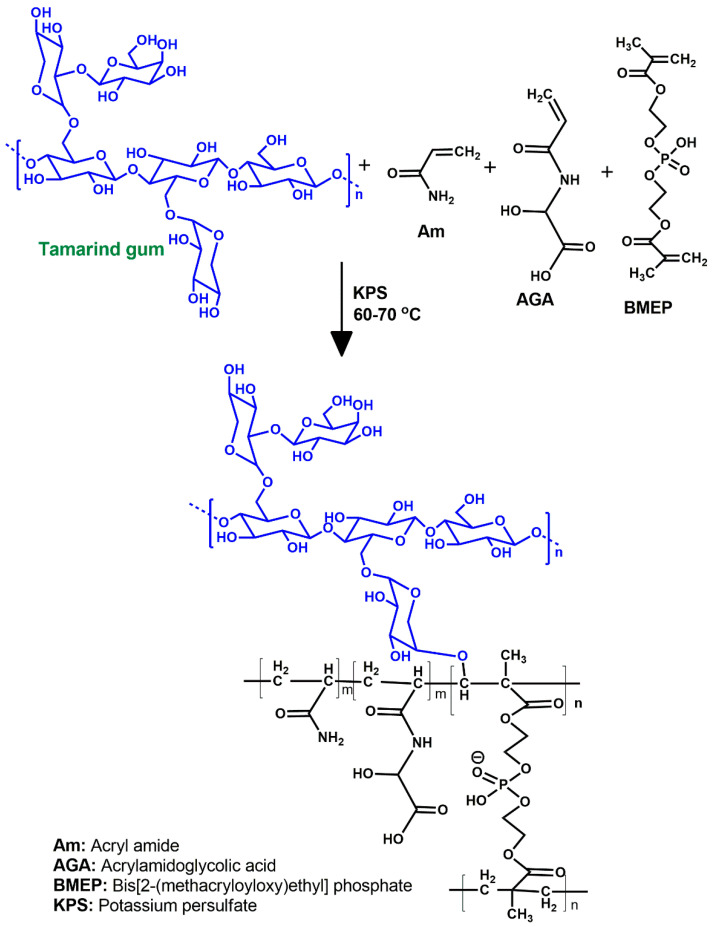
Plausible schematic chemistry of TMGA hydrogels.

Figure 1 shows the FTIR spectra of TMG polysaccharide, pure TMGA hydrogel, DOX, DOX-loaded TMGA hydrogel and TMGA-Ag. The pure tamarind gum (TMG) polysaccharide (Figure 1A) demonstrated a broad stretching vibration at 3416.32 cm^−1^ for aliphatic -O-H and 2920.95 cm^−1^ for -C-H groups. The pristine TMGA hydrogel spectrum is depicted in Figure 1B, with peaks observed at 1729.52 cm^−1^ and 1612.95 for (acid/amide) -C=O stretching vibration. The broad peak for TMG’s -O-H bending vibration was recorded at 1380.95 cm^−1^ [29,30]. Furthermore, peaks for P-O, P=O, and -C-O-C stretching vibrations emerged at 1216.19 and 1053 cm^−1^, confirming the existence of phosphate crosslinker (BMEP). Figure 2 depicts the pure medication DOX spectrum (C). DOX showed characteristic peaks at 3334.28 cm^−1^, corresponding to -O-H and N-H stretching vibrations, and another peak at 1720 cm^−1^, corresponding to -C=O stretching vibrations. The peak at 1592.38 cm^−1^ corresponds to unsaturated -C=C- stretching vibration and 1399.04 cm^−1^ corresponds to hydroxy group -O-H bending vibration. Figure 1D depicts the spectrum of a DOX-loaded TMGA hydrogel, with a characteristic peak at 3435.23 cm^−1^ corresponding to the -O-H functional moiety and a peak at 1667 cm^−1^ corresponding to the -CO-NH- functional moiety, and a peak at 1564 cm^−1^ corresponding to the unsaturated -C=C- stretching vibration of DOX. Figure 1E shows the FTIR spectrum of a green synthesis of TMGA hydrogel silver nanocomposite using aqueous leaf extract *Terminali bellirica*; the stretching vibrational peaks at 3425.12, 1612.89, 1389 and 1056.13 cm^−1^ reveal the phenolic moiety, amide and -C=O of hydrogels, respectively. The results show that TMG-based hydrogels can be successfully synthesized using simple free radical polymerization, DOX can be incorporated in TMGA hydrogels and silver nanoparticles can be produced in an environmentally safe manner. Figure 2 depicts a probable schematic chemistry of TMGA hydrogel.

### 2.2. UV–Visible Spectroscopy

**Figure 3:**
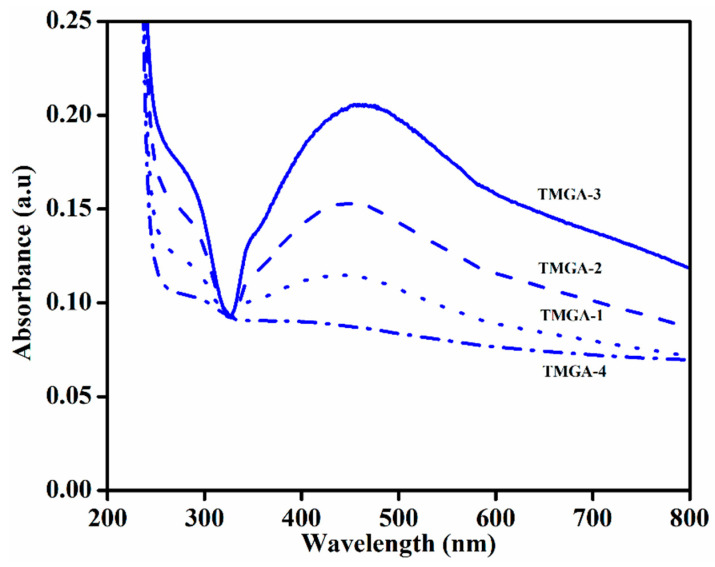
UV–Visible spectroscopy of TMGA-Ag hydrogels.

TGM-derived natural polysaccharide-based hydrogels were employed as templates for the green synthesis of solver nanoparticles from *Terminalia bellirica* leaf extract, an eco-friendly reducing agent. Essentially, the reduction of silver ions to silver nanoparticles is proven by a color change from light yellow to black at ambient temperature and a reaction time of 180 min, as shown in Figure 3. Significant absorption peaks were detected at 428–436 nm as a result of a quantum mechanical process known as surface plasmon resonance, which leads in the creation of silver nanoparticles within polymer networks with the help of polymer chains [31,32].

### 2.3. X-ray Diffraction Studies of TMGA Hydrogel

**Figure 4:**
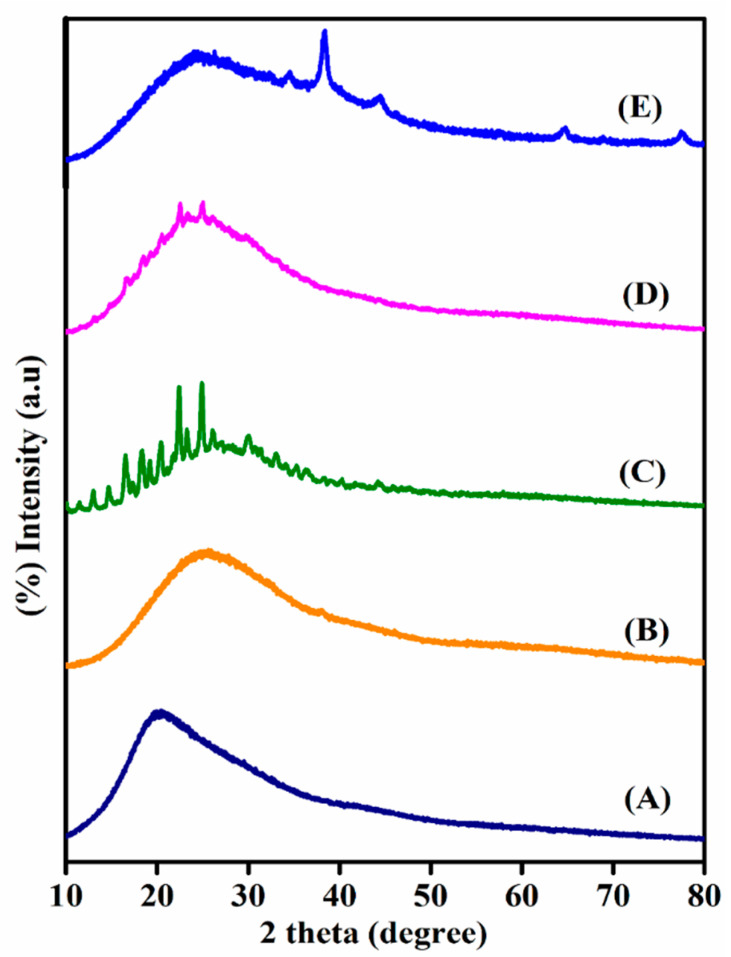
X-ray diffraction spectra of TMGA hydrogel. (**A**) pure tamarind gum, (**B**) pristine TMGA hydrogel, (**C**) pure DOX, (**D**) DOX-loaded TMGA hydrogel, (**E**) TMGA-Ag nanocomposites hydrogel.

XRD was used to analyze the crystalline behavior of pure tamarind gum, pristine TMGA hydrogel, pure DOX, DOX-loaded hydrogel and TMGA-Ag hydrogel; the diffractograms are shown in Figure 4. The XRD curves of pure tamarind gum and pristine TMGA hydrogel (Figure 4A,B) are not shown in any significant crystalline peaks, which may be due their amorphous behavior. However, the XRD curve of pure DOX (Figure 4C) shows significant crystalline peaks at two theta values: 17.30°, 23.24°, 25.24°, 32.40°, 35.25°, and 40.29°. The DOX-loaded TMGA hydrogel (Figure 4D) showed characteristic DOX peaks at two theta values: 17.52°, 19.53° and 25.29°, showing that DOX is physically present, chemically stable and molecularly distributed within the hydrogel network. The XRD pattern of the TMGA-Ag hydrogel (Figure 4E) showed typical peaks at 38.46°, 44.41°, 63.12° and 76.46° for planes (111), (200), (220) and (311), respectively [31,32]. These findings suggest that the silver nanoparticles found in the hydrogel network are face-centered cubic crystals in nature.

### 2.4. SEM and TEM Studies

**Figure 5:**
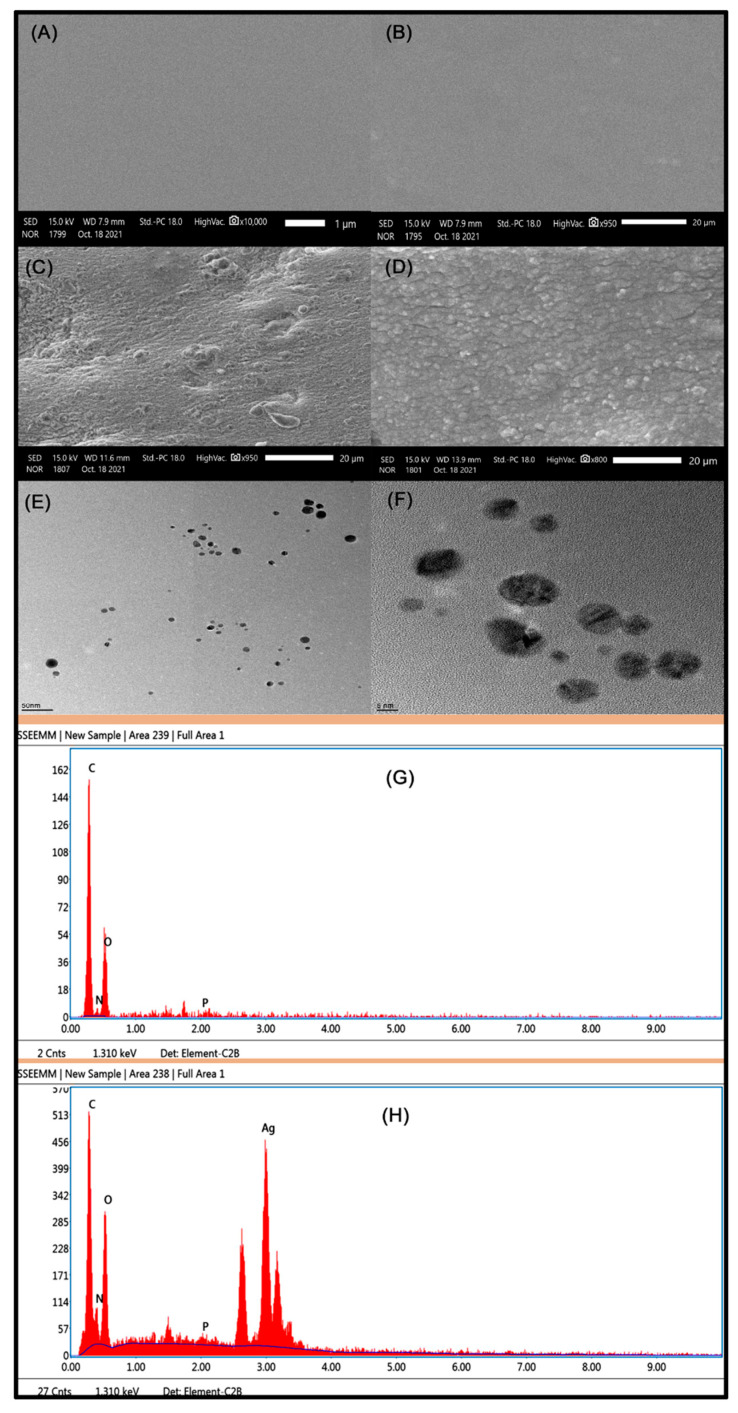
SEM and TEM images of TMGA hydrogels. (**A**,**B**) Pure hydrogel, (**C**) drug composite hydrogel, (**D**) TMGA-AgNP hydrogel and (**E**,**F**) TEM images of AgNPs, (**G**) TMGA hydrogel EDX spectrum, (**H**) TMGA-AgNPs EDX spectrum.

SEM and TEM are the most commonly employed techniques for the characterization of all types of materials to obtain quantitative data, i.e., morphology and particle size. The surface morphology and elemental analysis were analyzed for pristine TMGA hydrogel and DOX-loaded hydrogel, and TMGA-Ag hydrogels were examined with SEM and TEM; images are shown in Figure 5. Pristine hydrogel exhibited a smooth surface owing to hydrophilic behavior of functional moieties (hydroxyl and amide) present in the polymer network (Figure 5A,B). DOX-loaded TMGA hydrogel has a rough surface compared to pristine hydrogel (Figure 5C), which may be due to (1) the presence of DOX molecules in and outside the network, which are formed as debris at the time of dehydration; (2) the surface having a high surface area based on the synthesis process, resulting in the rough surface. Ag nanoparticles were produced by a green method using aqueous leaf extract to reduce the Ag^+^ ions. Figure 5D–F show the SEM and TEM images of PMGA-Ag hydrogel and Ag nanoparticles, respectively. TEM images show that that Ag nanoparticles are mainly spherical shapes and are formed without any aggregation. This suggests that the green synthesis of nanoparticles by green methods is good. The presence of silver elements and the size of Ag nanoparticles inside the hydrogel was examined with energy-dispersive X-ray spectroscopy (EDX). The EDX spectrums in Figure 5G,H are before and after the production of silver nanoparticles in TMGA hydrogels; in the TMGA-Ag hydrogel, an optical absorption peak was observed at 3.02 kV.

### 2.5. Swelling Studies

**Figure 6:**
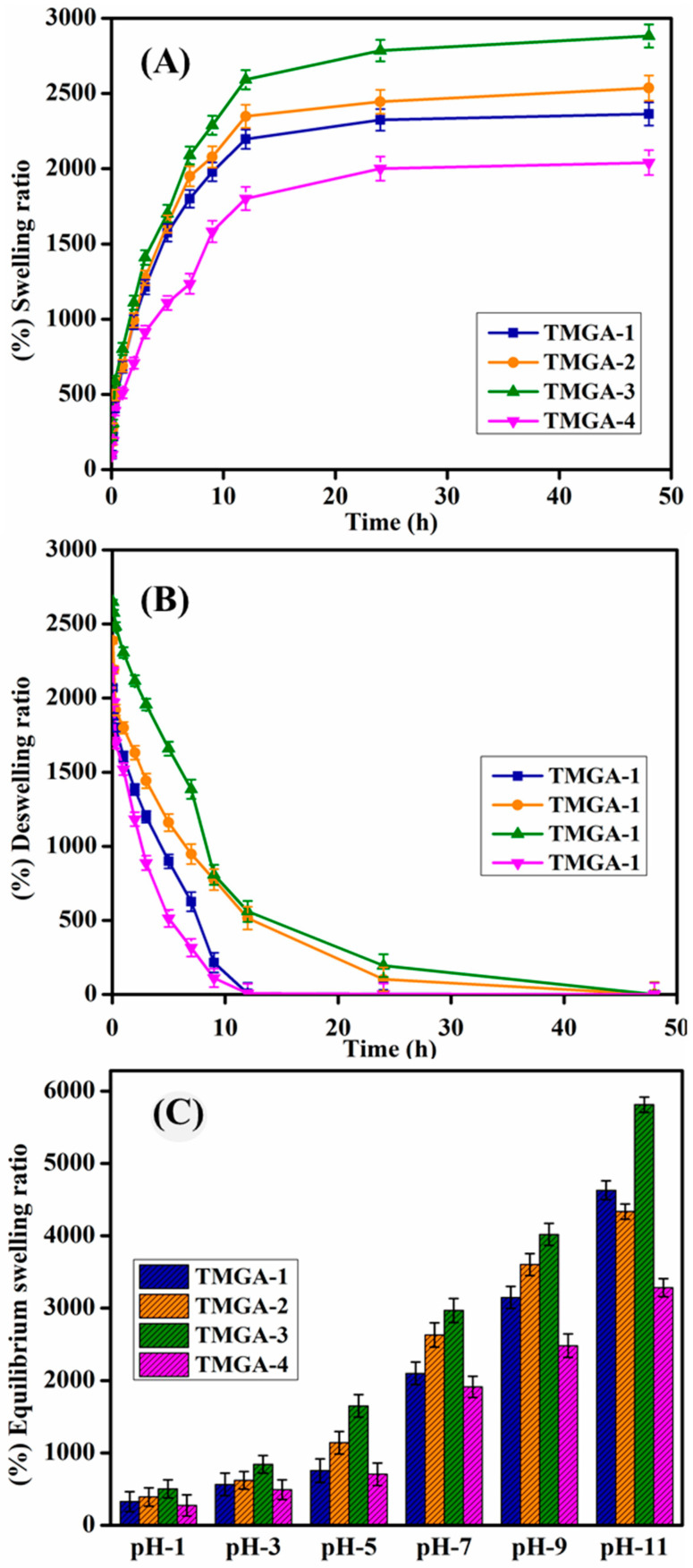
Swelling studies of TMGA hydrogels, (**A**) swelling, (**B**) deswelling, (**C**) pH-equilibrium swelling.

At room temperature, TMGA hydrogels were tested for (%) swelling ratio, (%) equilibrium swelling ratio (ESR), and pH–equilibrium swelling ratio (Table 1). Figure 6 depicts the swelling kinetics of TMGA hydrogels as well as pH-dependent equilibrium swelling. Among all of the formulations examined for swelling studies, TMGA-3 (2882) had the highest swelling ratio and TMGA-4 (2040) had the lowest. The results show that the swelling ratio of TMGA hydrogels increases when AGA concentration increases and reduces as BMEP concentration increases. pH-dependent equilibrium swelling experiments of TMGA hydrogels evaluated various pH media (1, 3, 5, 7, 9 and 11). The presence of functional groups such as -OH and -COOH, which is associated with more ionizable hydroxy and carboxylate ions in a basic environment, may explain why the percent of ESR was higher in the alkaline zone rather than the acidic.

For synthesized TMGA hydrogels, structural network parameters such as average molecular weight between crosslinkers (degree of crosslinking), polymer network volume fraction (*V*_2*s*_), mesh size (*ξ*), polymer crosslinker density (*υ_e_*) and solvent interaction parameter of polymer network chain (*χ*) were calculated using swelling results, and the results are shown in Table 1. With rising monomer (AGA) concentration and lowering crosslinker (BMEP) concentration, the average molecular weight between crosslinkers *M_c_* and *ξ* values rose. The polymer solvent interaction values of all the prepared hydrogels were approximately 0.52–0.54, indicating that the hydrogel network had good polymer solvent interactions. The data values for crosslinking density range from 0.11 to 0.54. These findings suggested that hydrogels become more hydrophilic as monomer content (AGA) increases and that hydrogels become softer and have a stronger network [28,30].

### 2.6. Encapsulation and In Vitro DOX Release of TMGA Hydrogels

**Figure 7:**
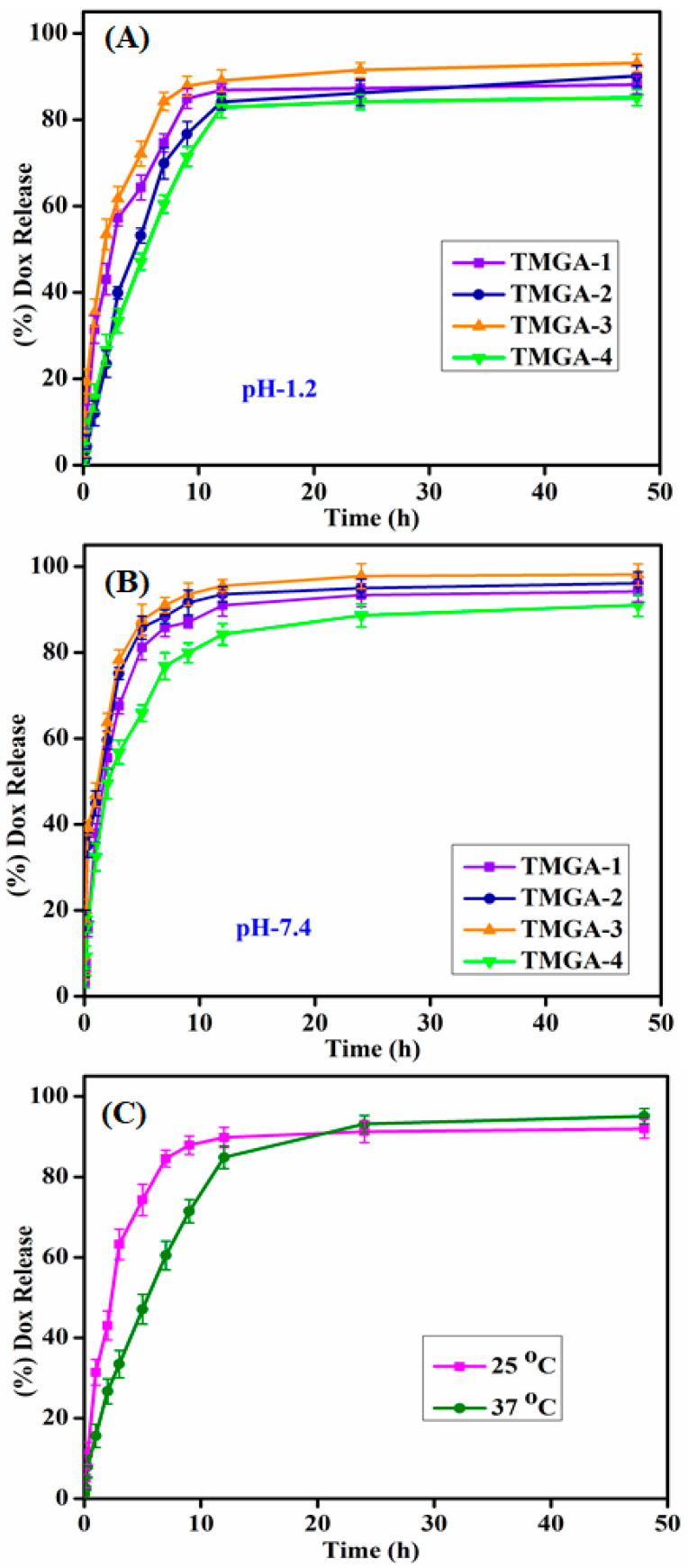
In vitro DOX release studies of TMGA hydrogel (**A**) pH-1.2 (**B**) pH-7.4, and (**C**) pH-7.4 at 25 and 37 °C.

The percentage of DOX encapsulation efficiency data of TMGA hydrogel are shown in Table 1; these values ranged between 31.86 ± 1.3 and 69.20 ± 1.2. The maximum (%) EE was observed in TMGA-3 and the minimum in TMGA-4; i.e., these values were influenced by monomers and crosslinkers in the hydrogel network. DOX-encapsulated TMGA hydrogels were tested using a tablet dissolution tester at 37 °C in various buffer solutions such as pH-1.2 (simulated gastric region) and pH-7.4 (simulated intestinal region). DOX release kinetics were shown against percentage of total drug release vs. time. In vitro DOX release studies were performed in pH 7.4 and 1.2 at 25 and 37 °C; release data are presented in Figure 7. DOX release results demonstrate that more drugs are released in pH 7.4 compared to pH 1.2, which may be due to the pH-responsive -COOH group of PAGA polymer chains (data shown in Table S1). This may be because TMGA hydrogels contain more hydrophilic groups and easily ionizable functionalities, such as carboxylic (-COOH) acid and hydroxyl (-OH); thus, the larger ionization polymer network in phosphate-buffered solution may be due to hydrogel expansion, and the ionic repulsion of COO^−^ and -OH functionality results in the formation of high-release DOX from the hydrogel network [33,34,35]. This behavior was clearly observed for TMGA-3, as the increasing concentration of monomer drug release was observed more and the increasing concentration of crosslinker drug release was observed less. Further, these results are supported by the equilibrium swelling data.

DOX release data fitted with various kinetics models, such as Higuchi square root, Hixson–Crowell cube root, zero-order, first-order and the Korsmeyer–Peppas equation, are shown in Table S1. The in vitro drug release kinetics mechanism fitted best to the Korsmeyer–peppas equation. The instance with a *n* < 0.45 polymer network followed Fickian diffusion, the second case between 0.45 < *n* < 0.89 corresponded to non-Fickian diffusion and an anomalous mechanism and in the third case, *n* = 1 totally non-Fickian and super case II drug release kinetics [21].

### 2.7. Cell Viability Assay and Cell Cycle Analysis of TMGA Hydrogel

**Figure 8:**
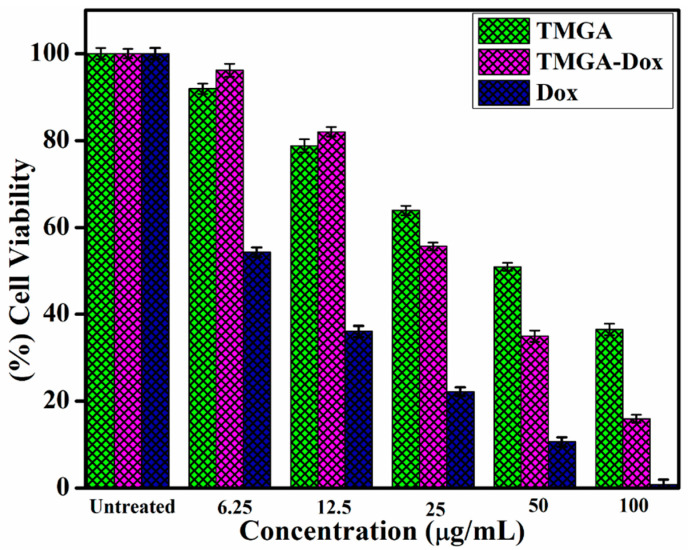
Cell viability studies of TMGA hydrogel, DOX and DOX-loaded gel after 24 h incubation of HCT-116 cell lines.

Cancer cells of the HCT-116 (human colorectal adenocarcinoma) cell line were incubated with different concentrations (6.25, 12.5, 25, 50 and 100) of pure TMGA, DOX and DOX-loaded TMGA hydrogel. Cell viability was investigated through MTT assays (Figure 8). From the MTT assay, IC_50_ values observed for pure TMGA hydrogel, pure DOX and DOX-loaded TMGA were 51.42 μg/mL, 6.67 μg/mL and 31.54 μg/mL, respectively. The pure TMGA hydrogel cell viability displayed higher IC_50_ values with lower cytotoxicity; DOX-loaded TMGA hydrogel exhibited significant cytotoxicity; compared to hydrogel formulations, free DOX is obviously more toxic [36,37,38].

**Figure 9:**
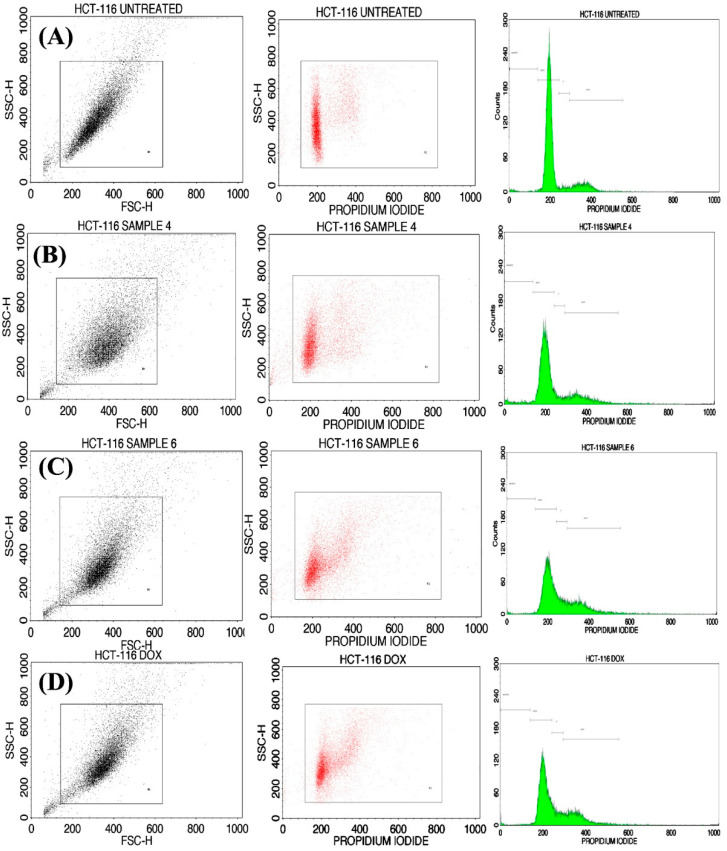
Cell cycle analysis of HCT116 cells (**A**) untreated, (**B**) TMGA hydrogel, (**C**) DOX-loaded TMGA hydrogel, (**D**) pure DOX.

The cell cycle analysis was carried out by staining treated and untreated cell lines with PI, which were subsequently examined by flow cytometry. The percentage of distribution cells in different stages of analysis for control and treated cells are presented in Figure 9. The cycle assay used HCT-116 cells analyzed by pure TMGA hydrogel, free DOX, and DOX-loaded TMGA hydrogel; apoptosis was investigated with flow cytometry. The untreated percentage of cell cycles corresponded to sub G0/G1, 1.46%, G0/G1 79.65%, S phase 3.67% and G2/M 15.22, The pure TMGA hydrogel percentage of cell apoptosis is as follows: sub G0/G1 1.43%, G0/G1 75.71%, S phase 35.11% and G2/M 17.39%, pure DOX percentage of cell apoptosis given data sub G0/G1 1.36%, G0/G1 57.2%, S phase 13.92% and G2/M 26.98%, DOX-loaded TMGA hydrogel percentage of cell apoptosis given data sub G0/G1 2.93%, G0/G1 62.33%, S phase 12.13% and G2/M 21.68%, respectively; the results indicate that the HCT116 cells underwent either apoptosis or necrosis.

### 2.8. Anti-Microbial Studies of TMGA-Ag Nanocomposites

**Figure 10:**
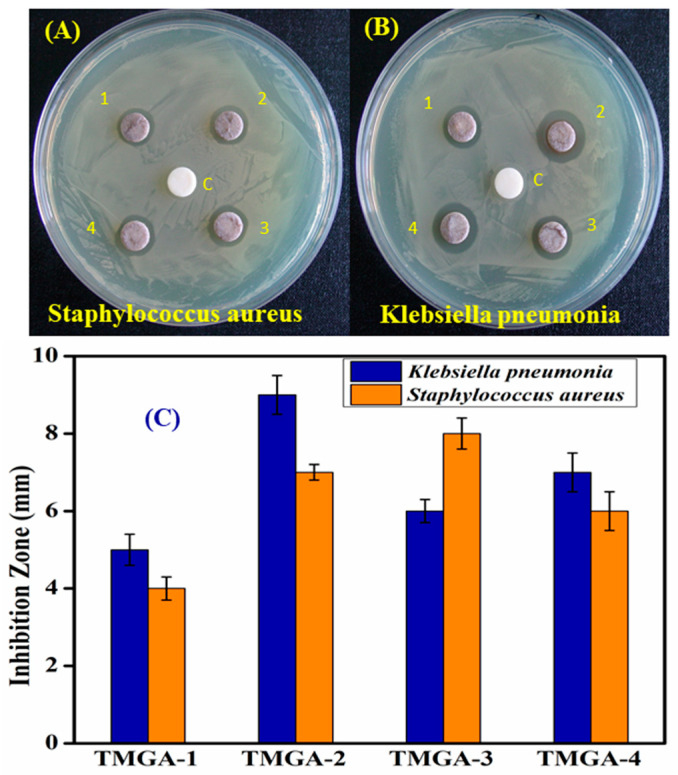
Photographs of Antimicrobial activity of TMGA hydrogels against *Staphylococcus aureus* (**A**) *Klebsiella pneumoniae* (**B**) and zone inhibition (**C**).

The TMGA-Ag nanocomposite hydrogel was developed for the inactivation of multi-drug-resistant (MDR) bacteria, including both Gram-positive and Gram-negative bacteria (Figure 10). Zone inhibition of TMGA-Ag against *Klebsiella pneumonia* was 6.0 mm, 9.0 mm, 6.0 mm and 7.0 mm, and zone inhibition of *Staphylococcus aureus* was 4.0 mm, 7.0 mm, 8.0 mm and 6.0 mm. The TMGA-Ags inhibited both Gram-positive and negative pathogen bacteria in the zone. The anionic nature of the generated TMGA-Ags absorbed at the cell membrane’s surface resulted in the formation of Ag nanoparticle diffusion, which led to the inactivation of MDR bacteria [39,40].

## 3. Conclusions

➢ In the current study, a simple free radical polymerization was used to synthesize an affordable, safe, biocompatible and biodegradable tamarind gum polysaccharide and acylamidoglycolic acid-based hydrogel (TMGA).
➢ TMGA hydrogels were successfully utilized as templates for the fabrication of silver nanoparticles (13.4 ± 3.6 nm in diameter) via a green method using aqueous leaf extract of *Teminalia bellirica* as a reducing agent. TMGA-Ag nanocomposite hydrogel was identified with UV–Visible absorption spectra λ_max_ 425–432 nm.
➢ FT-IR, UV–Vis., XRD, SEM, EDS and TEM were used to examine the chemical structure and functional groups of the pure TMGA hydrogel and TMGA-Ag nanocomposite hydrogels, as well as their morphological features of the hydrogel network.
➢ Doxorubicin hydrochloride (DOX) was encapsulated into the hydrogels with an encapsulation effectiveness of 69.20 ± 1.2—DOX was successfully entrapped in the hydrogel network. After 48 h in pH 7.4 at 37 °C, the maximum percentage DOX release was 93.12 percent.
➢ In vitro DOX release of TMGA hydrogels was investigated using various kinetic models such as zero-order, first-order, Higuchi and Hixson–Crowell, and Korsmeyer–Peppas equations, as well as swelling kinetics, pH equilibrium, and network characteristics.
➢ The TMGA hydrogel completely followed the non-Fickian diffusion and was the best fitted with the model Korsemeyer–Peppas transport mechanism.
➢ The TMGA-Ag nanocomposite hydrogels efficiently inactivated MDR microorganisms *Staphylococcus aureus* and *Klebsiella pneumoniae*.
➢ Finally, the created hydrogel could be employed not only for the pH-triggered release of DOX but also for any other hydrophilic chemotherapeutic agent and antibacterial applications based on the findings of this investigation.

## 4. Materials and Methods

### 4.1. Material and Methods

Bis(2-(methacryloyloxyethyl) phosphate (BMEP), acylamidoglycolic acid (AGA) and potassium persulphate (KPS) were purchased from Sigma-Aldrich (Milwaukee, WI, USA). Sodium hydroxide and hydrochloride were purchased from S.D Fine Chemical Limited (Mumbai, India). Acrylamide, silver nitrate and potassium dihydrogen phosphate (KH_2_PO_4_) were purchased from Merck. Doxorubicin Hydrochloride (DOX) were received from LC laboratories (Woburn, MA, USA). Tamarind seeds were collected from the local market. Double distilled water (DDW) water was used for all experiments.

### 4.2. Isolation of Tamarind Gum (TMG) Polysaccharide

TMG was isolated as per the procedure published elsewhere [13]. Briefly, the raw seeds of the tamarind fruit were collected and washed with DDW to remove the pulp and kernel, and 30 g of washed seeds was then crushed into small pieces and ground into a fine powder. The powder was put in a 1000 mL glass beaker containing 600 mL of water and boiled in water at 60–70 °C. Constant stirring was maintained to attain a viscous solution, and the solution was filtered and washed twice with acetone and then with hexane. The precipitate was collected via centrifugation. The acetone and hexane were removed at 50 °C in a vacuum oven, and the filtrate TMG polysaccharide was stored in a refrigerator until further use.

### 4.3. Synthesis of Tamarind Gum Based Hydrogels

Aqueous TGM solution (2% *w*/*v*) was prepared and 5.0 mL of the solution was transferred to 50 mL beaker; to this, required quantities of acrylamide and acylamidoglycolic acid (AGA) monomers were added. Then, an initiator, potassium persulfate (1 mL 10% aqueous solution) and a crosslinker, bis(2-(methacryloyloxy) ethyl phosphate (BMEP) (2.0 mL of 10% aqueous solution) were added and stirred for 6 h to obtain a homogenous solution. This reaction mixture was transferred to a thermally controlled water bath (50 °C), and in a 30 min reaction, the solution was converted to transparent gel; however, the reaction continued for 4 h to ensure a complete reaction. The formed hydrogels (Figure S1) were then immersed in DDW to remove trace quantiles of unreacted chemical species and every 24 h, the water was changed for 4 days. Finally, the hydrogels were dried at 40 °C in a hot air oven until a constant weight was reached. Digital photograph of synthesized TMGA hydrogels are presented in Figure S2.

### 4.4. Green Synthesis of Ag-NPs Hydrogel Network

A total of 300 mg of dried TMGA hydrogel was weighed and transferred into double distilled water. The swollen hydrogels were then transferred into 25 mL of AgNO_3_ aqueous solution (5 mM), and then, hydrogels were allowed to equilibrate for 30 h to ensure the maximum silver ions were in the hydrogel network. In this process, a large amount of silver ions was exchanged from the solution into the hydrogel network by hydrophilic groups of the hydrogel chain. The remaining metal ions occupied the free space of the hydrogel network through the ion exchange process. This Ag^+^-ion-loaded hydrogel was transferred to another beaker containing 15 mL of aqueous leaf extract of Teminalia bellirica, which resulted in the formation of the silver nanoparticles from silver ions within 2 h. The color of hydrogels changed from light transparent to black, which confirmed the formation of silver nanoparticles. The developed TMGA silver nanocomposite hydrogel (Figure S1) was washed in double distilled water then dried in a hot air oven at 40 °C.

### 4.5. Swelling Studies of TMGA Hydrogels

TMGA hydrogel swelling kinetic studies were performed in double distilled water at 30 °C. A total of 100 mg of dried hydrogel was placed in air-tight bottles containing 50 mL of double distilled water. The swollen hydrogel weight was measured after carefully wiping the surface-adhered water using a soft tissue paper. The equilibrium swelling was performed in different pH solutions (1, 3, 5, 7, 9 and 11). The (%) swelling ratio (% SR) and (%) equilibrium swelling ratio (% ESR) were calculated based on the weights of the dried and swollen hydrogels using the following equation:

(1) $$\left[\%\right]\;\mathrm{Swelling}\;\mathrm{ratio}\;\left(\mathrm{SR}\right)=\left[\frac{w_{s}-w_{d}}{w_{d}}\right]\times 100$$

(2) $$\left[\%\right]\mathrm{Equlibrium}\;\mathrm{swelling}\;\mathrm{ratio}\;\left(\mathrm{ESR}\right)=\left[\frac{w_{e}-w_{d}}{w_{d}}\right]\times 100$$

where w_s_, w_d_ and w_e_ are the swollen, dried and equilibrium weights of the hydrogel, respectively.

### 4.6. Network Parameters of TMGA Hydrogels

TMGA hydrogel network parameters were calculated based on equilibrium-swelling data using different equations [19]. The molecular mass between the crosslinks (*M_c_*) was calculated by the following equation:

(3) $${\bar{M}}_{c}exp=-\frac{\left(1-\frac{2}{\emptyset }\right)v_{1}\rho_{2}{\left(v_{2r}\right)}^{\frac{3}{2}}{\left(v_{2m}\right)}^{\frac{1}{3}}}{\ln\left(1-v_{2m}\right)+v_{2m}+\chi v_{2m}{}^{2}}$$

(4) $$v_{2r}=\left[1+\frac{\left(\frac{w_{r}}{w_{d}}-1\right)\rho_{2^{-1}}}{\rho_{1}}\right]$$

(5) $$v_{2m}=\left[1+\frac{\left(\frac{w_{s}}{w_{d}}-1\right)\rho_{2^{-1}}}{\rho_{1}}\right]$$

The Flory–Huggins interaction (polymer–solvent) parameter (*χ*) was calculated as follows:

(6) $$\chi =\frac{1}{2}+\frac{v_{2m}}{3}$$

The mesh size (*ξ*) and crosslinking density (*υ_e_*) network parameters were calculated with the following equation:

(7) $$\xi =0.071\phi^{\frac{-1}{3}}{\left(M_{c}\right)}^{\frac{1}{2}}$$

(8) $$v_{e}=\rho \sqrt{M_{c}}$$

### 4.7. Drug Loading and Encapsulation Efficiency

DOX, a chemotherapeutic agent, was used as the model drug to examine the drug release characteristics of TMGA hydrogel. The DOX loading and encapsulation efficiency of TMGA hydrogel was determined through the physical equilibrium swelling method. The dried TMGA hydrogel (about 200 mg) was transferred to 25 mL of DOX solution (1 mg/mL), equilibrated for 24 h at room temperature, then removed and dried at 40 °C. The dried DOX-loaded hydrogel (10 mg) was placed into phosphate-buffered solution (PBS) and left for 48 h for the DOX to be removed completely. The residual DOX solution was analyzed using a LAB INDIA ultraviolet–visible spectrophotometer (model: UV-3092) at λ_max_ 489 nm. The drug loading and encapsulation efficiency were calculated using the following equations [20].

(9) $$\left[\%\right]\;\mathrm{Drug}\;\mathrm{loading}=\left[\frac{\mathrm{Dox}\;\mathrm{loaded}\;\mathrm{TMGA}\;\mathrm{hydrogel}}{\mathrm{Amount}\;\mathrm{of}\;\mathrm{TMGA}\;\mathrm{hydrogel}}\right]\times 100$$

(10) $$\left[\%\right]\;\mathrm{EE}=\left[\frac{\mathrm{Actual}\;\mathrm{loading}\;\mathrm{of}\;\mathrm{TMGA}\;\mathrm{hydrogels}\;}{\mathrm{Theoritical}\;\mathrm{loading}\;\mathrm{of}\;\mathrm{TMGA}\;\mathrm{hydrogel}}\right]\times 100$$

### 4.8. In Vitro Drug Release Studies of TMGA Hydrogels

For the TMGA drug-loaded hydrogels, drug release was carried out through a tablet dissolution tester, which was thermo stated and consisted of 8 basket LAB INDIA USP tablet dissolution testers (model: DS-8000). The dissolution rotation per speed was constantly 100 rpm, and drug release rates were measured at 25 and 37 °C. In vitro drug release studies were performed by two different conditions: pH 1.2 (gastric region) and 7.4 (intestinal) phosphate-buffered solutions. The baskets were filled with 500 mL of buffer solution and each drug loaded, roughly 100 mg sample was dried at regular time intervals; in each sample, 5.0 mL was taken out and replaced with 5.0 mL of fresh buffer solution. The amount of DOX released was analyzed through using an ultraviolet–visible spectrophotometer (LAB-INDIA, UV-3092) at a fixed wavelength at 481 nm. The samples measurements were collected using triplicate data estimation for standard deviation. The formulation TMGA-3 hydrogel experiments were also performed at 25 °C in pH 7.4 phosphate-buffered solution.

### 4.9. DOX Release Kinetic Parameters

The results of DOX release studies of TMGA hydrogel fitted with different kinetic release models (Table S2), i.e., Higuchi square root, Hixson–Crowell cube root, zero-order, first-order, and the Korsmeyer–Peppas equation, were calculated with the following equations [21]:

(11) Mt=Kh12t

(12) $$Q=Q_{o}-K_{o}t$$

(13) $$lnQ=lnQ_{o}-K_{1}t$$

(14) Q13=Q013K0C

(15) $$\frac{M_{t}}{M_{\infty }}=kt^{n}$$

where *K_h_*: Higuchi rate constant, *Q*: amount of drug released at time *t*, *Q*_0_: amount of drug released at time *t* = 0; *K*_1_: first-order rate constant, *K*_0_: zero-order rate constant, *K_C_*: the drug release constant Hixson-Crowell cube root law. *M_t_*: drug release at time t, *M_∞_*: drug release at time ∞; and *k*: diffusion coefficient and *n*: diffusion exponent.

### 4.10. Cell Viability and Cell Cycle Studies of TMGA-Ag Hydrogels

The cell viability of HCT-116 cell lines was tested using the MTT assay with pure TMGA hydrogel, DOX-loaded hydrogel and pure DOX. Flow Cytometry (BD Bioscience, San Jose, CA, USA) was used to examine the HCT-116 cell cycle studies of pure TMGA hydrogel, DOX-loaded hydrogel and pure DOX. (1-2).

### 4.11. Antimicrobial Studies of TMGA-Ag Hydrogels

The TMGA-Ag hydrogel was tested for antimicrobial activity of Staphylococcus aureus and Klebsiella pneumonia through the disk diffusion method [19]. The first nutrient agar media was prepared by mixing 5 g of peptone, 3 g of beef extract and 5 g of sodium chloride (NaCl) in double distilled water, the pH of the mixture was adjusted to 7, and finally, 15 g of agar was added to the solution. Then, the agar medium was sterilized and autoclaved at a pressure of 15 lbs and a temperature of 121 °C for 1 h. This medium was transferred into Petri dishes in a laminar air flow chamber. An aqueous 4 mg/4 mL solution of the sample was prepared. A 25 µL sample was then added to paper discs (6 mm) on the surface of the bacterial plates and incubated at 37 °C for 16 h. The zone of inhibition formed around the disc was measured.

### 4.12. Characterization of TMGA-Ag Hydrogels

FTIR spectroscopy (Bruker Alpha-II, Eco-ATR, Billerica, MA, USA) was performed to detect the various functional groups and chemical structures of the TMGA hydrogel network with a scanning range between 4000 and 600 cm^−1^. The green synthesis of silver nanoparticles in the hydrogel network was characterized by UV-Visible spectroscopy (UV-Vis, LAB INDIA, UV-3092, Mumbai, India) with a scanning range between 200 and 800 nm. X-ray diffraction (Rigaku, mini flex 600, Tokyo, Japan) of pure TMGA hydrogel and DOX-loaded hydrogel was performed at a scanning speed of 5^°^ /min using Ni-filtered Cu-Kβ radiation in a range up to 10–80° 2θ. The shape size and surface morphology studies of pure TMGA gel, DOX-loaded TMGA hydrogel and TMGA-Ag hydrogels were characterized by SEM (JEOL-IT500A, Tokyo, Japan) with acceleration voltages of 0.3 to 30 kV. The hydrogel was cut into about 20 mg samples, then coated with ultrafine gold coater and placed on the carbon stub acerated speed of voltage 0.2 to 30 kV. The morphology of TMGA hydrogel samples were studied using JEOL-IT500A, Tokyo, Japan. The EDX (energy dispersive X-rays) spectrum analysis of the TMGA drug composite and TMGA-Ag nanocomposite hydrogel samples were analyzed by JEOL-IT500A, Tokyo, Japan. Ag-NP-embedded TMGA hydrogels were characterized by field emission transmission electron microscopy (TEM, FEI, Tecnai F20) that operated at 80 kV. The briefly colloidal aqueous TMGA-Ag nanocomposites hydrogel solution was casted on a carbon-coated 300-mesh copper grid and left for 30 min. The image was captured at different ranges of magnification.

## Figures and Tables

**Table 1:** Feed composition of TMGA hydrogel, % equilibrium swelling (% ESR), % encapsulation efficiency (% EE) and network parameters.

Sample Code	TMG (2%) (mL)	Am (g)	AGA (g)	BMEP (2%) (*w*/*v*) (mL)	KPS 10% (*w*/*v*) (mL)	% ESR ESR	% EE	Network Parameters				
								*χ*	*M_c_*	*ξ*	*ø*	*υ_e_*
TMGA-1	5.0	0.5	0.5	2.0	1.0	2364 ± 1.0	39.61 ± 1.0	0.5456	29,653	39.451	12.659	0.4552
TMGA-2	5.0	0.5	1.0	2.0	1.0	2536 ± 0.7	56.56 ± 0.8	0.5325	36,039	50.125	11.478	0.1256
TMGA-3	5.0	0.5	1.5	1.0	1.0	2882 ± 1.2	69.20 ± 1.2	0.5238	61,464	66.265	10.355	0.1125
TMGA-4	5.0	0.5	1.0	3.0	1.0	2040 ± 0.9	31.86 ± 1.3	0.5243	21,782	32.236	15.119	0.5426

## Supplementary Materials

The following are available online at https://www.mdpi.com/article/10.3390/gels7040237/s1. ^1^H NMR of TMG polysaccharide (Figure S1), digital photograph of synthesized TMGA hydrogels (Figure S2), in vitro drug release data of TMGA hydrogel (Table S1) and various drug release kinetic parameters (Table S2).
