# Supplementary material for: Synthesis of Novel Tamarind Gum-co-poly(acrylamidoglycolic acid)-Based pH Responsive Semi-IPN Hydrogels and Their Ag Nanocomposites for Controlled Release of Chemotherapeutics and Inactivation of Multi-Drug-Resistant Bacteria
Source: Gels. 2021 Nov 27;7(4):237. doi: 10.3390/gels7040237 (PMC8701875; doi:10.3390/gels7040237)
Supplement: Supplementary file 1 [file gels-07-00237-s001.zip › gels-1465440-supplementary.pdf]

Supplementary Materials

Synthesis of Novel Tamarind Gum-co-poly(acrylamidoglycolic acid)-Based pH Responsive Semi-IPN Hydrogels and Their Ag Nanocomposites for Controlled Release of Chemotherapeutics and Inactivation of Multi-Drug-Resistant Bacteria

Kasula Nagaraja [1], Kummari S.V. Krishna Rao [1,*], Sunmi Zo [2,3], Sung Soo Han [2,3], Kummara Madhususdana Rao [2,3,*]

1 Polymer Biomaterial Design and Synthesis Laboratory, Department of Chemistry, Yogi Vemana University, Kadapa 516005, Andhra Pradesh, India; nagarajakasula33@gmail.com
2 School of Chemical Engineering, Yeungnam University, 280 Daehak-ro, Gyeongsan 38541, Gyeongbuk, Korea; sunmizo@ynu.ac.kr (S.Z.); sshan@yu.ac.kr (S.S.H.)
3 Research Institute of Cell Culture, Yeungnam University, 280 Daehak-ro, Gyeongsan 38541, Gyeongbuk, Korea
* Correspondence: ksvkr@yogivemanauniversity.ac.in (K.S.V.K.R.); msraochem@gmail.com (K.M.R.); Tel.: +91-9704278890 (K.S.V.K.R)

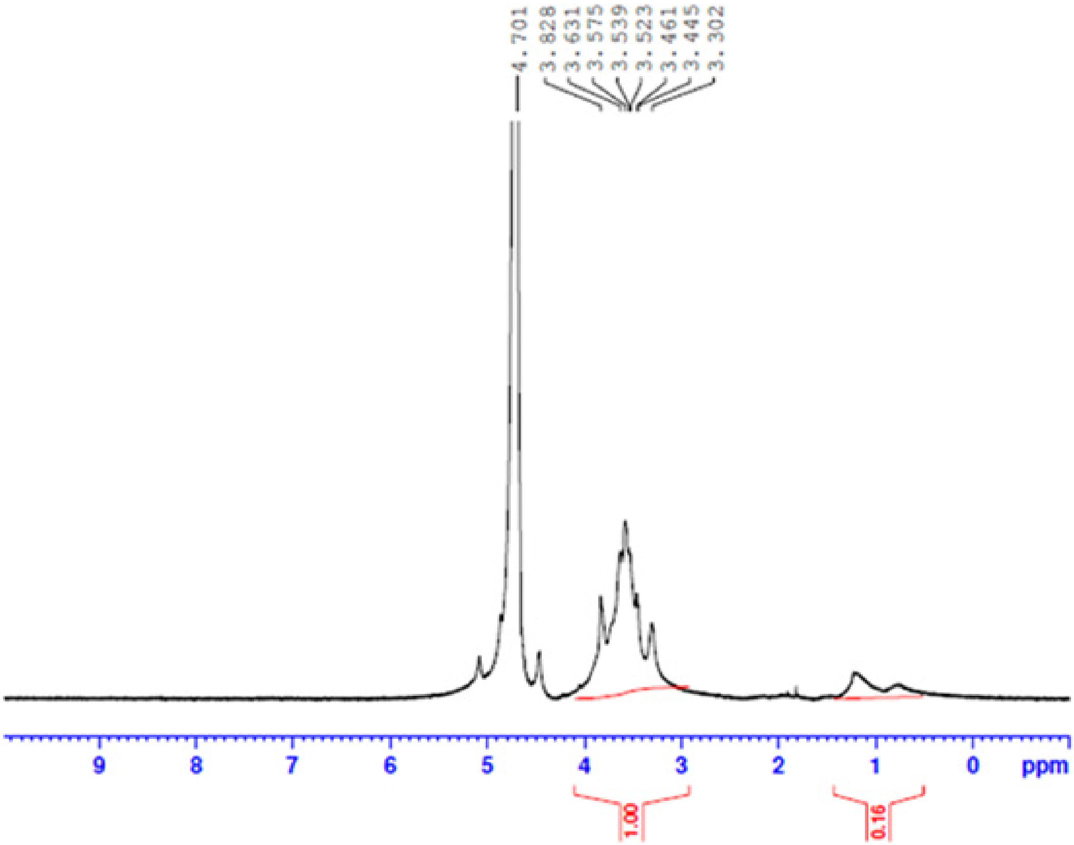

Figure S1: ^{1}HNMR spectroscopy of pure tamarind gum polysaccharide

The TMG polysaccharide was isolated from TM seeds and confirmed by ^{1}HNMR spectroscopy using D_2O as a solvent. The characterization peaks for TM showed a narrow region at 4.4 to 3.3 ppm indicating the anomeric proton peaks of the glucose, xylose, and galactose backbone.

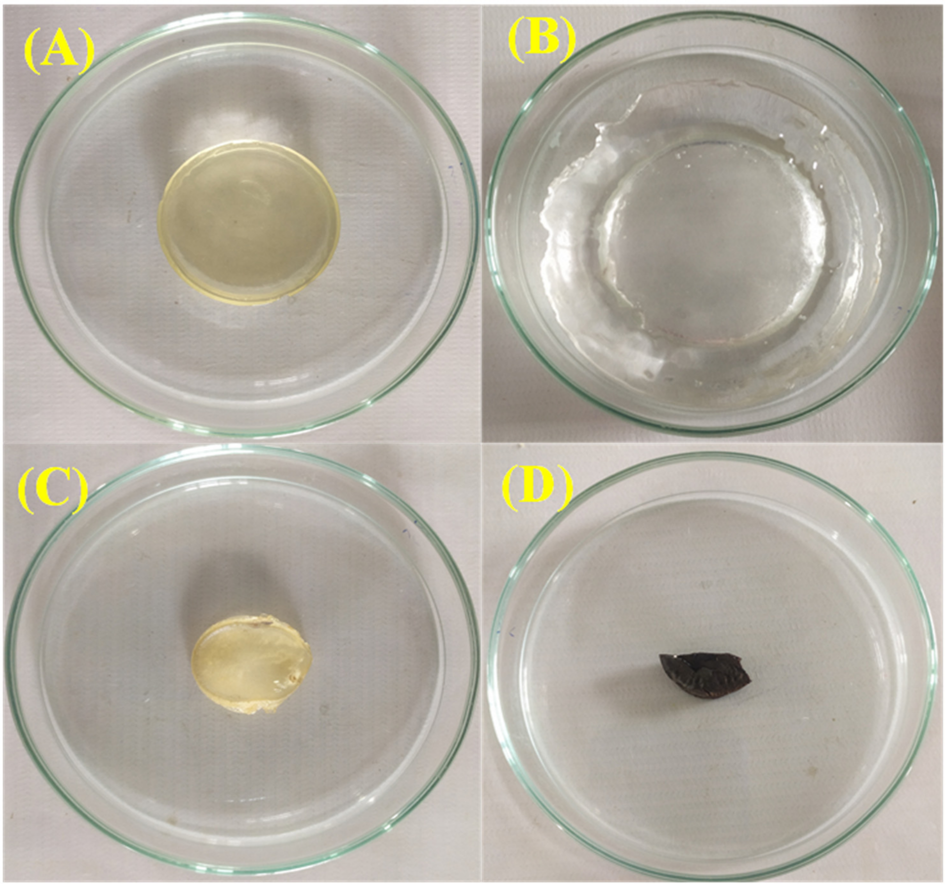

Figure S2: Digital photographic images of TMGA hydrogels (A) Fresh gel (B) Swollen gel (C) dried gel (D) TMGA-Ag hydrogel

Table S1: In-vitro drug release data of TMGA hydrogel

Time (h)	pH-1.2 (%)		pH-7.4 (%)	
	**TMGA-3**	**TMGA-4**	**TMGA-3**	**TMGA-4**
3	35.31	26.68	48.88	32.40
5	53.41	33.42	63.51	49.58
7	61.74	47.09	75.25	65.91
12	72.12	71.46	84.23	84.23
24	89.27	84.16	95	88.68
48	93.12	85.12	97.72	91.02

Table-S2: Drug release kinetics of various kinetics models

Sample code	Zero order		First order		Higuchi		Hixson-Crowell		Korsmeyer- peppas					
									pH-1.2			pH-7.4		
	R	K_0	*R*	K_1	*R*	K_h	*R*	K_{hc}	*k*	*n*	*r*	*k*	*n*	*r*
**TMGA-1**	0.982	2.215	0.978	2.145	0.991	0.102	0.963	1.215	2.51	0.98	0.977	2.33	0.64	0.984
**TMGA -2**	0.965	4.325	0.961	3.215	0.997	0.295	0.980	1.784	1.33	0.79	0.994	3.56	0.95	0.984
**TMGA -3**	0.989	1.849	0.988	1.478	0.974	0.380	0.991	1.235	2.35	0.67	0.981	1.47	0.91	0.991
**TMGA -4**	0.978	3.457	0.977	1.213	0.964	1.264	0.974	0.984	2.14	0.78	0.995	4.12	0.76	0.986
